# A novel force transduction pathway from a tension sensor to the gate in the mechano-gating of MscL channel

**DOI:** 10.3389/fchem.2023.1175443

**Published:** 2023-06-06

**Authors:** Yasuyuki Sawada, Takeshi Nomura, Boris Martinac, Masahiro Sokabe

**Affiliations:** ^1^ Department of Physiology, Nagoya University Graduate School of Medicine, Nagoya, Japan; ^2^ Institute of Materials Innovation, Institutes of Innovation for Future Society, Nagoya University, Nagoya, Japan; ^3^ International Cooperative Research Project, Solution Oriented Research for Science and Technology (ICORP/SORST), Cell Mechanosensing, Japan Science and Technology Agency (JST), Nagoya, Japan; ^4^ Molecular Cardiology and Biophysics Division, Mechanosensory Biophysics Laboratory, Victor Chang Cardiac Research Institute, Darlinghurst, NSW, Australia; ^5^ School of Human Science and Environment, University of Hyogo, Himeji, Japan; ^6^ Human Information Systems Laboratories, Kanazawa Institute of Technology, Hakusan, Ishikawa, Japan

**Keywords:** mechanosensitive channel, *E-coli*, MscL, gating mechanism, patch clamp, molecular dynamics, force transduction, membrane tension

## Abstract

The bacterial mechanosensitive channel of large conductance MscL is activated exclusively by increased tension in the membrane bilayer. Despite many proposed models for MscL opening, its precise mechano-gating mechanism, particularly how the received force at the tension sensor transmits to the gate remains incomplete. Previous studies have shown that along with amphipathic *N*-terminus located near the cytoplasmic surface of the membrane, Phe78 residue near the outer surface also acts as a “tension sensor,” while Gly22 is a central constituent of the “hydrophobic gate.” Present study focused on elucidating the force transmission mechanism from the sensor Phe78 in the outer transmembrane helix (TM2) to the gate in the inner transmembrane helix (TM1) of MscL by applying the patch clamp and molecular dynamics (MD) simulations to the wild type MscL channel and its single mutants at the sensor (F78N), the gate (G22N) and their combination (G22N/F78N) double mutant. F78N MscL resulted in a severe loss-of-function, while G22N MscL caused a gain-of-function channel exhibiting spontaneous openings at the resting membrane tension. We initially speculated that the spontaneous opening in G22N mutant might occur without tension acting on Phe78 residue. To test this hypothesis, we examined the (G22N/F78N) double mutant, which unexpectedly exhibited neither spontaneous activity nor activity by a relatively high membrane tension. To understand the underlying mechanism, we conducted MD simulations and analyzed the force transduction pathway. Results showed that the mutation at the tension sensor (F78N) in TM2 caused decreased interaction of this residue not only with lipids, but also with a group of amino acids (Ile32-Leu36-Ile40) in the neighboring TM1 helix, which resulted in an inefficient force transmission to the gate-constituting amino acids on TM1. This change also induced a slight tilting of TM1 towards the membrane plane and decreased the size of the channel pore at the gate, which seems to be the major mechanism for the inhibition of spontaneous opening of the double mutant channel. More importantly, the newly identified interaction between the TM2 (Phe78) and adjacent TM1 (Ile32-Leu36-Ile40) helices seems to be an essential force transmitting mechanism for the stretch-dependent activation of MscL given that substitution of any one of these four amino acids with Asn resulted in severe loss-of-function MscL as reported in our previous work.

## 1 Introduction

Mechanosensitive (MS) channels are expressed in eukaryotic and prokaryotic cells and play critical roles in a variety of physiological functions such as touch sensation, sound detection, gravity perception and osmoregulation (Hamill and Martinac, 2001; Kung et al., 2010). Among them the best studied MS channels are the bacterial mechanosensitive channel of small conductance (MscS) and mechanosensitive channel of large conductance (MscL), which protect bacteria from the cell lysis upon hypo-osmotic shock by releasing small osmolytes and water, thus both serving as “safety valves” (Levina et al., 1999).

**FIGURE 1:**
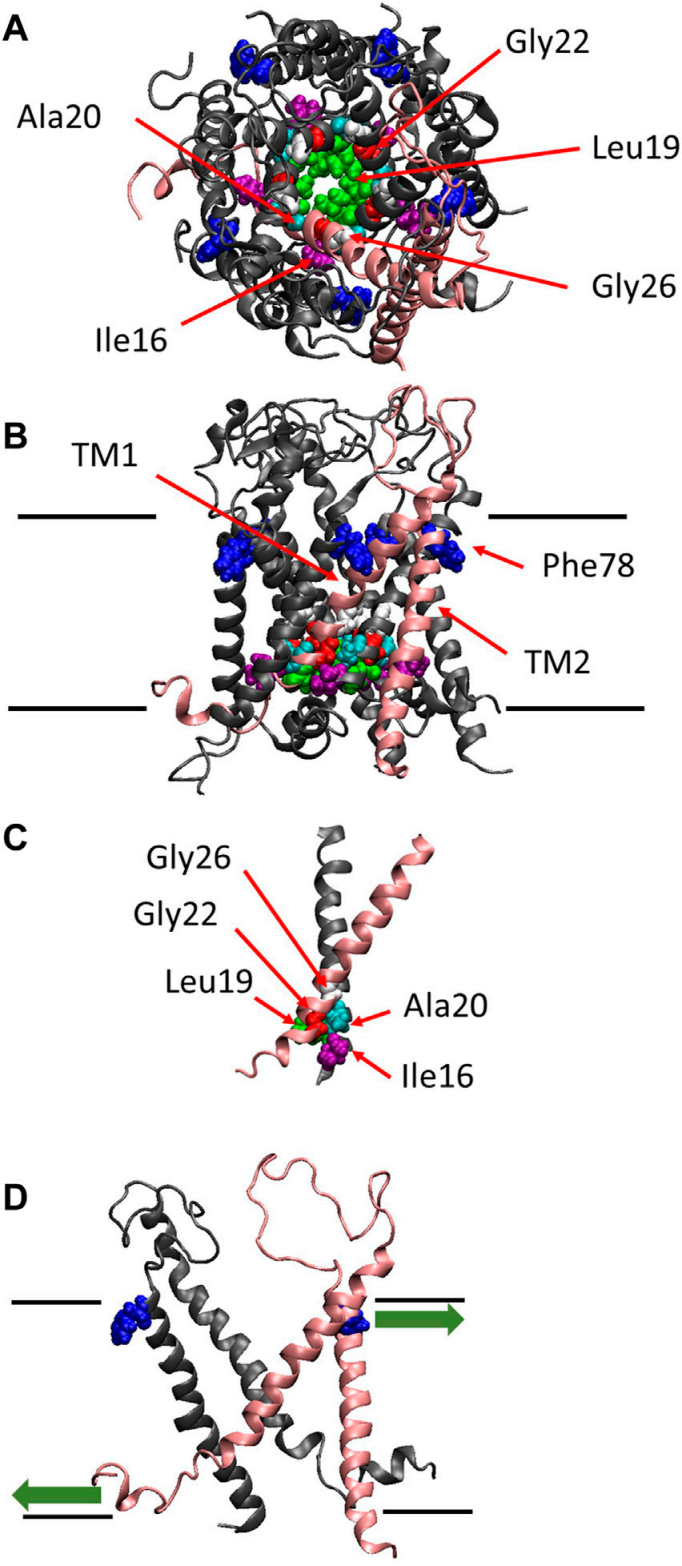
The crystal structure of MscL. Top **(A)**, side **(B)** views and the crossing (interacting) portion formed by the two TM1 helices **(C)** of the structural model of *E. coli* MscL (Sukharev et al., 2001). **(D)** Side view with two specific subunits representing the direction of pulling force at Phe78 in TM2 and N-terminal domain. A single subunit is highlighted in pink, and Ile16, Leu19, Ala20, Gly22, Gly26 and Phe78 are depicted in space-filling format in purple, green, cyan, red, white and blue, respectively. The approximate location of lipid membrane is shown by the parallel lines in the side view.

MscL from *Escherichia coli* (Eco-MscL) is a homopentamer of a subunit constituted of 136 amino acids with two transmembrane α-helices named TM1 and TM2 in the inner cytoplasmic membrane (Sukharev et al., 1994). An X-ray crystal structure of the closed or nearly-closed state of the MscL homologue from *Mycobacterium tuberculosis* (Tb-MscL) was first resolved at 3.5 Å, which has revealed that five TM1 α-helices line the pore including hydrophobic gate near the cytoplasmic end of the pore, while TM2 α-helices interact with membrane lipids and both the N- and C-termini are located in the cytoplasm (Chang et al., 1998) (Figures 1A, B). The single subunit has a molecular mass of ∼15 kDa and conductance of the channel pentamer is ∼3 nS (Sukharev et al., 1994).

The channel retains mechanosensitivity when the purified proteins are reconstituted into artificial lipid membranes (Sukharev et al., 1993; Häse et al., 1995; Blount et al., 1996; Sukharev et al., 1999; Nomura et al., 2012; Nomura et al., 2015), indicating that MscL is opened directly by bilayer tension. Replacing one of seven hydrophobic amino acids near the lipid-water interface in the outer leaflet of the bilayer with a hydrophilic amino acid residue (Asn) causes a loss of function MscL, suggesting that they contribute to sensing membrane tension, leading to channel opening (Yoshimura et al., 2004). Among the seven amino acid residues phenylalanine 78 (Phe78) has been shown to play a significant role in tension sensing by a molecular dynamics study (Sawada et al., 2012). It is located near the lipid-water interface of the outer leaflet of the cytoplasmic membrane and is directly acted upon by tension in the lipid bilayer (Yoshimura et al., 2004; Sawada et al., 2012) (Figure 1B). The three amino acids (Ile16, Leu19 and Ala20) in TM1 interact with the amino acids (Gly22 and Gly26) in the neighboring TM1 helix, which form the most constricted hydrophobic part of the channel pore called gate (Figure 1C). Previous random and scanning mutagenesis studies have shown that hydrophobic residues of the five amino acids (Ile16, Leu19, Ala20, Gly22, and Gly26), located at the cytoplasmic half of TM1 form the channel gate, with the closed state is stabilized by hydrophobic interactions between the residues on the neighboring TM1 subunits, called “hydrophobic lock” (Ou, et al., 1998; Yoshimura et al., 1999; Moe et al., 2000; Batiza et al., 2002; Iscla et al., 2004; Levin and Blount, 2004) (Figure 1C). It has also been reported that replacing Gly22, a key residue lining the gate of MscL, with Asn produces a gain of function MscL, in which spontaneous channel openings are observed even in the absence of membrane stretch both in giant spheroplasts and liposomal membranes (Yoshimura et al., 1999; Yoshimura et al., 2008).

More recently, it has been reported that the amphipathic N-terminal helix tightly interacts with membrane lipids and plays a crucial role in MscL opening upon stretching the membrane (Iscla et al., 2008; Iscla and Blount, 2012; Bavi et al., 2016). Apparently, MscL senses membrane tension at both the cytoplasmic and periplasmic side of the membrane. The tension-dependent force-from-lipids (Teng et al., 2015; Martinac and Kung, 2022) seems to act on both the Phe78 residue and the N-terminal helix in opposite directions (Figure 1D), thus contributing to the opening of the channel gate in a cooperative manner, by tilting transmembrane helices and opening the MscL channel. The N-terminal helix is linked to the gate region within TM1 via Gly14, enabling the force to act on the gate directly. However, it remains unclear how the force acting on Phe78 in TM2 may influence the channel gate in TM1, i.e., how the force is transmitted from Phe78 to the residues forming the channel gate.

The present study aimed to answer the question by analyzing and comparing the gating behaviors of GOF mutant (G22N), LOF mutant (F78N), double mutant (G22N/F78N) and wild type (WT) MscL channels using patch clamp and molecular dynamics simulations. Our results suggest that at least two contact sites between the TM2 helix and an immediately neighboring segment of the TM1 helix significantly contribute to tilting of the TM1 helix by transmitting the force sensed at Phe78 in TM2 to the gate residues including the Gly22 in TM1 of the adjacent subunit.

## 2 Materials and methods

### 2.1 Strains

*E. coli* strains MJF455 (*∆mscL::Cm*, *∆yggB*) (Levina et al., 1999) was used to host MscL channels used in patch clamp experiments.

### 2.2 Mutagenesis

Site-directed mutagenesis was performed by the megaprimer method as described previously (Yoshimura et al., 1999) and verified by DNA sequencing with the CEQ 2000XL DNA Analysis System (Beckman Coulter, Fullerton, CA).

### 2.3 Spheroplast preparation and electrophysiology

*E. coli* spheroplasts were prepared as described in (Blount et al., 1999) Briefly, MJF455 cells were grown for 1.5 h in the presence of cephalexin (final concentration 0.06 mg/mL), and IPTG (isopropyl-β-D-thiogalactoside) was added (final concentration 1 mM) to induce *mscL* expression. The induction time was 10 min and then collected by centrifugation and digested with lysozyme (final concentration 0.15 mg/mL). Recordings were performed by the inside-out patch clamp method as described previously (Yoshimura et al., 1999). The pipette solution contained 200 mM KCl, 90 mM MgCl_2_, 10 mM CaCl_2_, and 5 mM HEPES (pH 6.0), while the bath solutions contained additional 300 mM sucrose to stabilize the spheroplasts. All recordings were performed at +20 mV pipette voltage. Currents were amplified with an Axopatch 200B amplifier (Axon Instruments, Foster City, CA), filtered 2–10 kHz via a 4-pole low pass Bessel filter and sampled at 5–25 kHz with a Digidata 1322A interface using pCLAMP 10 software (Molecular Devices, Sunnyvale, CA). Negative pressure was applied to the patch pipette using a syringe-generated suction or a High-Speed Pressure Clamp-1 apparatus (HSPC-1; ALA Instruments, Farmingdale, NY). A pressure gauge (PM 015R, World Precision Instruments, Sarasota, FL) was used to measure the pressure throughout the experiments.

### 2.4 Molecular dynamics simulations

#### 2.4.1 System setup for simulation

In our computational study, we utilized the models of *E. coli* MscL in a closed state with S1 helices running parallel to the cytoplasmic membrane surface, proposed in our recent study (Sawada and Sokabe, 2015). The MscL model was embedded in a fully hydrated POPC bilayer and then solvated to place TIP3P water model on both the periplasmic and cytoplasmic sides of the membrane (Grubmüller, 1996). After the solvating step, we confirmed that no water molecules penetrated into the most constricted part of the pore formed by hydrophobic amino acid residues (Leu19, Gly22, and Val23). Finally, the system constructed above was minimized for 10,000 steps with a fixed backbone of the channel and then equilibrated for 50 ns with unrestrained (351 lipids, 66 potassium and 71 chloride ions, ∼23,000 water molecules, ∼125,000 atoms in total).

#### 2.4.2 Modeling of mutant MscLs

F78N, G22N, and G22N/F78N double mutant MscLs were modeled based on the wild-type (denoted as WT) model using the Mutate Residue utility in VMD (Humphrey et al., 1996). The amino acid residue(s) which was substituted with Asn were selected first and changed its side chain to that of Asn automatically in the utility. Then energy minimization was performed for 10,000 steps in each system after the modeling to remove bad contacts, especially around the substituted residue and the following equilibrium calculations (simulations without externally applied force (stretch) to the membrane) were performed for 50 ns During this process, a resting tension is naturally present in the membrane as shown in the pressure profile of the membrane, in which it has two distinct characteristic peaks corresponding to the strong negative pressure around the glycerol moiety in the outer and inner leaflets, respectively, (Cantor, 1997). After 50 ns of the equilibration simulation, we checked that the root mean square deviation (RMSD) value for the Cα atoms of the mutant MscL became nearly constant for relaxation of the model.

#### 2.4.3 Computational details

All MD simulations were carried out with NAMD (ver. 2.9), utilizing the CHARMM27 force field (Darden et al., 1993; Reiling and Schlenkrich, 1996; MacKerell et al., 1998; Kalè et al., 1999). Both the equilibrium and the channel opening simulations were set as an NPT ensemble at 310K, under 1 atm. The particle-mesh Ewald method for long-range electrostatics estimation was used with a 12 Å cutoff for short-range electrostatic and van der Waals forces. Periodic boundary conditions were employed with the dimensions of 120 × 120 × 100 Å. Visualization of states, molecular modifications and analysis were done in VMD using the embedded Tcl script language (Humphrey et al., 1996). In all simulations, a negative pressure at 150 dyn/cm was generated only in the lateral axis in the membrane while a constant pressure of 1 bar was set in the *z*-direction for 5 ns. Note that the timescale for the steered transition is much shorter than estimated in real experiments (–10 µs), but the simulation provides sufficient time for thermal relaxation of side chains along the opening path.

#### 2.4.4 Analysis

Analysis of the interaction energy was conducted by using a NAMDENERGY plug-in in VMD (Humphrey et al., 1996). The minimum pore radius of MscL was calculated by the HOLE program using a spherical probe (Smart et al., 1996). In the present study, pore radii were calculated in the plane where amino acid Leu19 is located, which has been suggested to be the most constricted part of the pore. Change in the tilt angle of TM1 inner helix was calculated based on the change in the coordinates of TM1 helix between the beginning and after 50 ns of equilibrium simulation.

## 3 Results

### 3.1 Electrophysiology of GOF (G22N) and LOF (F78N) mutants

**FIGURE 2:**
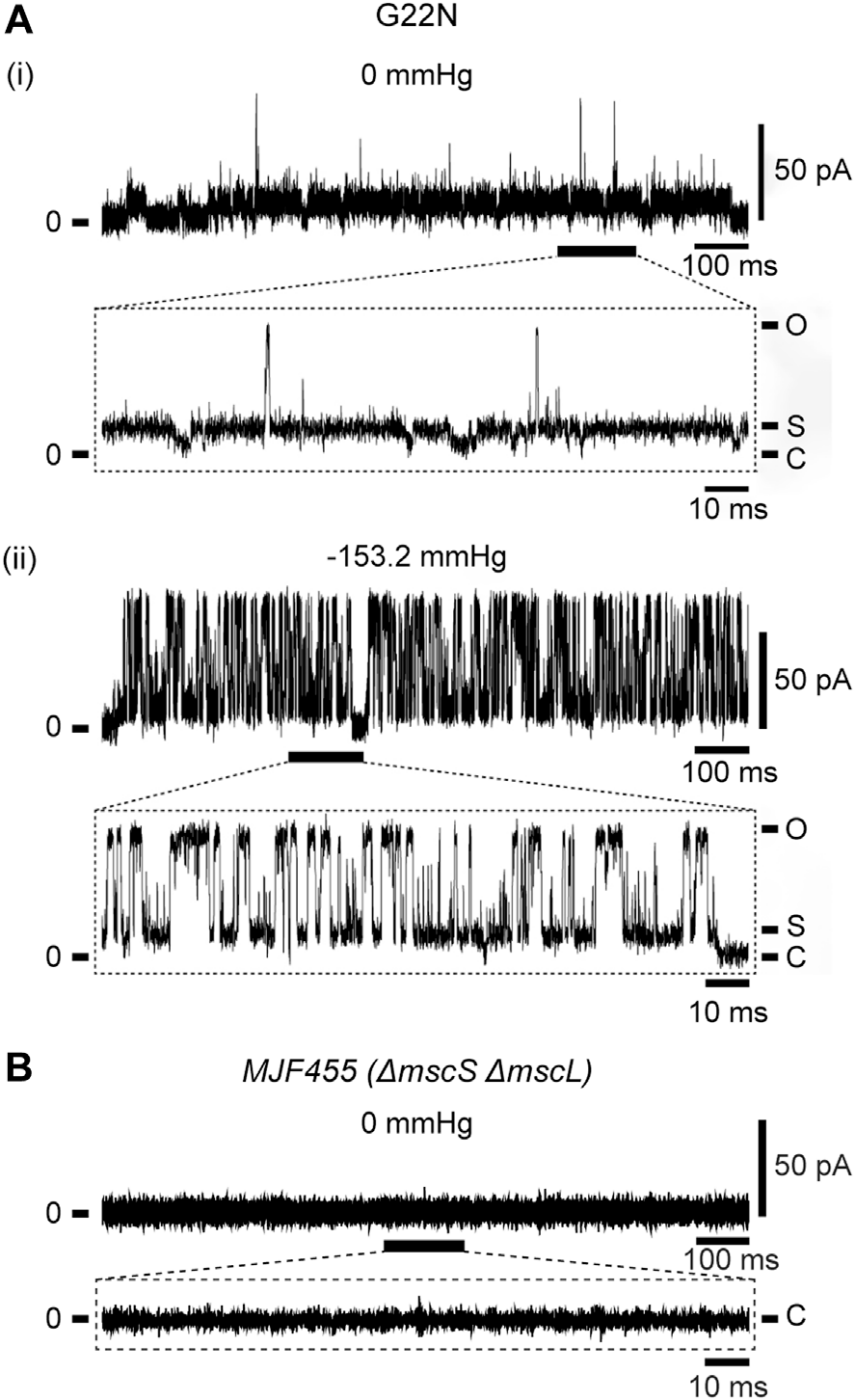
Spontaneous single-channel openings of G22N MscL expressed in MJF455 cells **(A)**. Representative current traces of G22N MscL in the absence **(i)** or presence **(ii)** of negative pressure (−153.2 mmHg) applied in the pipette. The current trace recorded from MJF455 cells harboring empty plasmid in the absence of negative pressure in the patch pipette **(B)**. The insets show the magnification of a part of G22N MscL and MJF455 current traces, respectively. The pipette potential was held at +20 mV.

As described in Introduction, Gly22 has been regarded as one of the key residues constituting the gate of MscL (Yoshimura et al., 1999; Yoshimura et al., 2004; Yoshimura et al., 2008; Petrov et al., 2011) and its replacement with hydrophilic residues (Asp, Asn or Glu) causes poor cell growth and spontaneous channel openings. We confirmed in this study that G22N MscL expressed in MJF455 cells indeed showed spontaneous channel openings in the absence of membrane stretch (Figure 2Ai) and a decreased threshold to membrane stretch (negative pressure in the pipette) (Figure 2Aii), which is entirely consistent with previous studies (Yoshimura et al., 1999; Yoshimura et al., 2008). Although G22N MscL showed spontaneous channel openings, there is a possibility that it might reflect endogenous WT MscL channel activity. To check for this possibility we performed patch-clamp recordings from MJF455 cells, and observed no spontaneous channel openings (Figure 2B).

**FIGURE 3:**
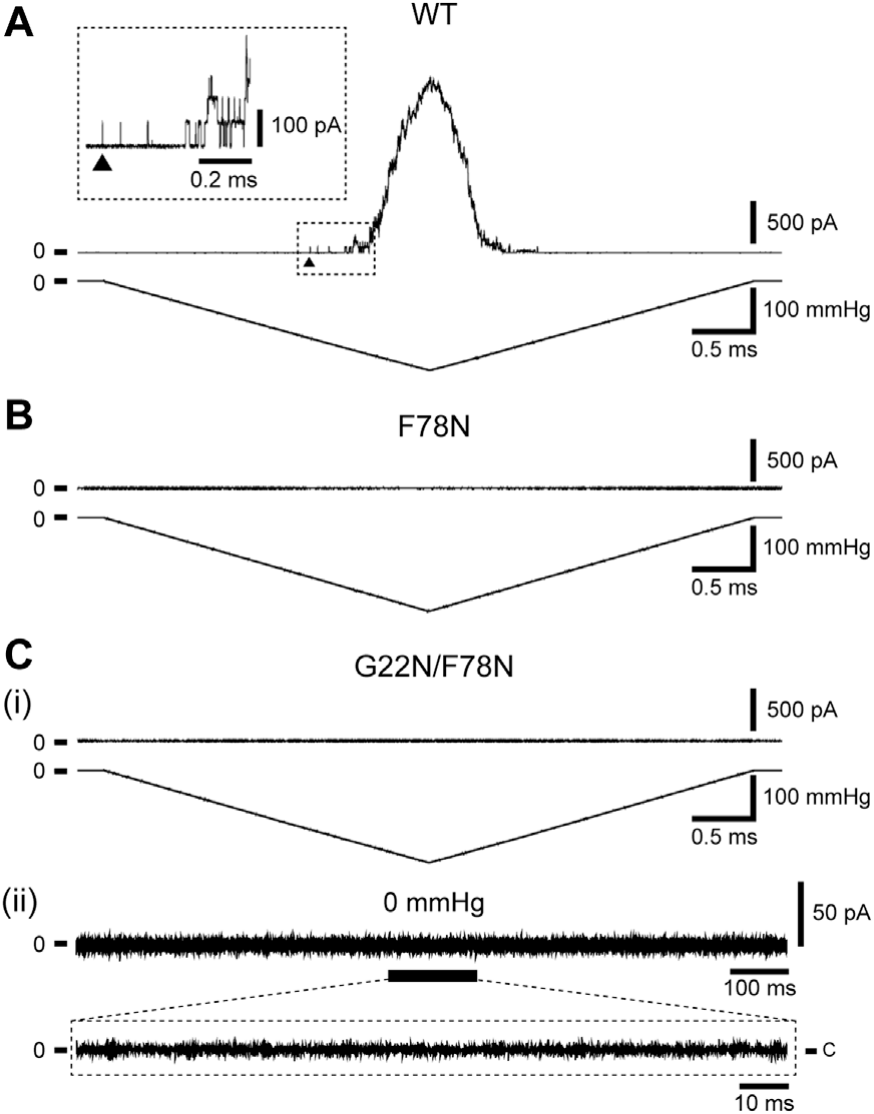
Typical current traces of WT **(A)**, F78N **(B)** and G22N/F78N **(C)** MscLs expressed in MJF455 cells. Representative current traces of G22N/F78N in the presence **(i)** or absence **(ii)** of negative pressure applied to the pipette. In each panel, the membrane current (*top*) and the negative pressure (*bottom*) are shown. The insets show the explanation of a portion of WT and G22N/F78N current traces. The arrowhead shows the first opening of WT MscL. Pipette potential was +20 mV.

Increase in the lipid bilayer tension will pull on Phe78 to tilt TM2 and TM1 α-helices, leading to the channel opening (Figure 1D), which is supported by the fact that Phe78 forms a strong hydrophobic interaction with the lipid bilayer (Sawada et al., 2012), where aromatic side chain may act as an anchor to interact with lipids (Domene et al., 2003). Phe78 is highly conserved in bacterial MscL channels (≥80%) (Sukharev et al., 2001; Balleza and Lagunas, 2009) and is located at the periplasmic rim based on a structural model of the closed channel (Sukharev et al., 2001). Figures 3A, B show typical current traces of Wild type and F78N MscL channels, respectively. Compared with WT, F78N MscL shows no channel activities even at a highly negative pressure in the patch pipette, which is consistent with our previous results (Yoshimura et al., 2004), as well as the hypothesis that Phe78 acts as a membrane tension sensor indispensable for MscL channel opening.

### 3.2 Electrophysiology of double mutant (G22N/F78N) and functional assay

Detailed mechanisms how the sensed force at the Phe78 tension sensor transmits to the gate and contributes to the opening of MscL is still poorly understood. To shed light on this issue, we investigated how the tension sensor Phe78 is coupled to the critical gate residue Gly22 by using the double mutant (G22N/F78N) MscL. As reported previously, G22N mutation destabilizes the “hydrophobic gate” and the mutant channel exhibits spontaneous openings in the absence of membrane stretch (Yoshimura et al., 1999; Yoshimura et al., 2008). We speculated that G22N mutation might bypass tension sensing process and induce spontaneous channel openings just by destabilizing the hydrophobic lock of the gate. Therefore, we hypothesized that the double mutation (G22N/F78N) would not affect the spontaneous channel opening. Surprisingly enough, the patch clamp results showed that not only stretch-dependent activation (Figure 3Ci) but also spontaneous channel openings (Figure 3Cii) were completely abolished in the double mutant MscL. This implies that even the spontaneous openings may require an interaction between the mutated gate component and the tension sensor Phe78. Mutation of the tension sensor (F78N) should interfere with this interaction to inhibit the spontaneous channel openings. We explored this possibility by performing all atom MD simulations. In the following sections, results from MD simulations on the gating behaviors of the three mutants along with WT MscL are shown.

### 3.3 MD simulations of equilibration process in wild-type and mutant MscL models

To explore the detailed mechanisms underlying the experimental results in the present study, we performed all atom MD simulations. As an initial step, simulations of the equilibrium process of ∼50 ns were performed with the three MscL mutants, F78N, G22N, and G22N/F78N, as well as WT MscL.

**FIGURE 4:**
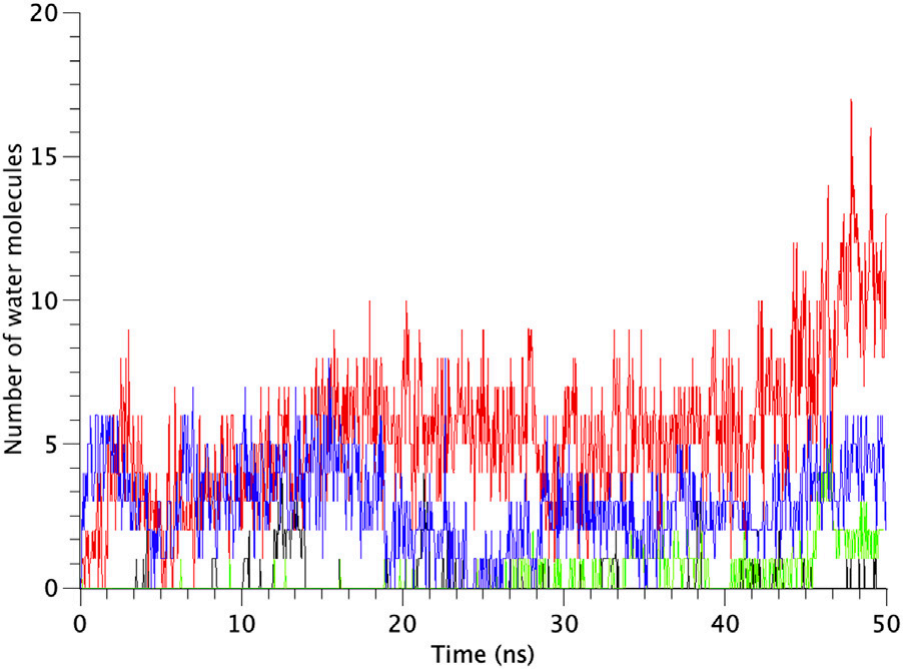
Time-course of the number of water molecules in the gate region of WT, G22N, F78N and G22N/F78N MscLs during the 50-ns equilibrium simulations, shown as black-, red-, green- and blue-colored lines, respectively. The water molecules are identified as those in the most constricted part of the pore formed by the amino acids Leu19 to Val23, which is defined as the hydrophobic portion of the gate.

**FIGURE 5:**
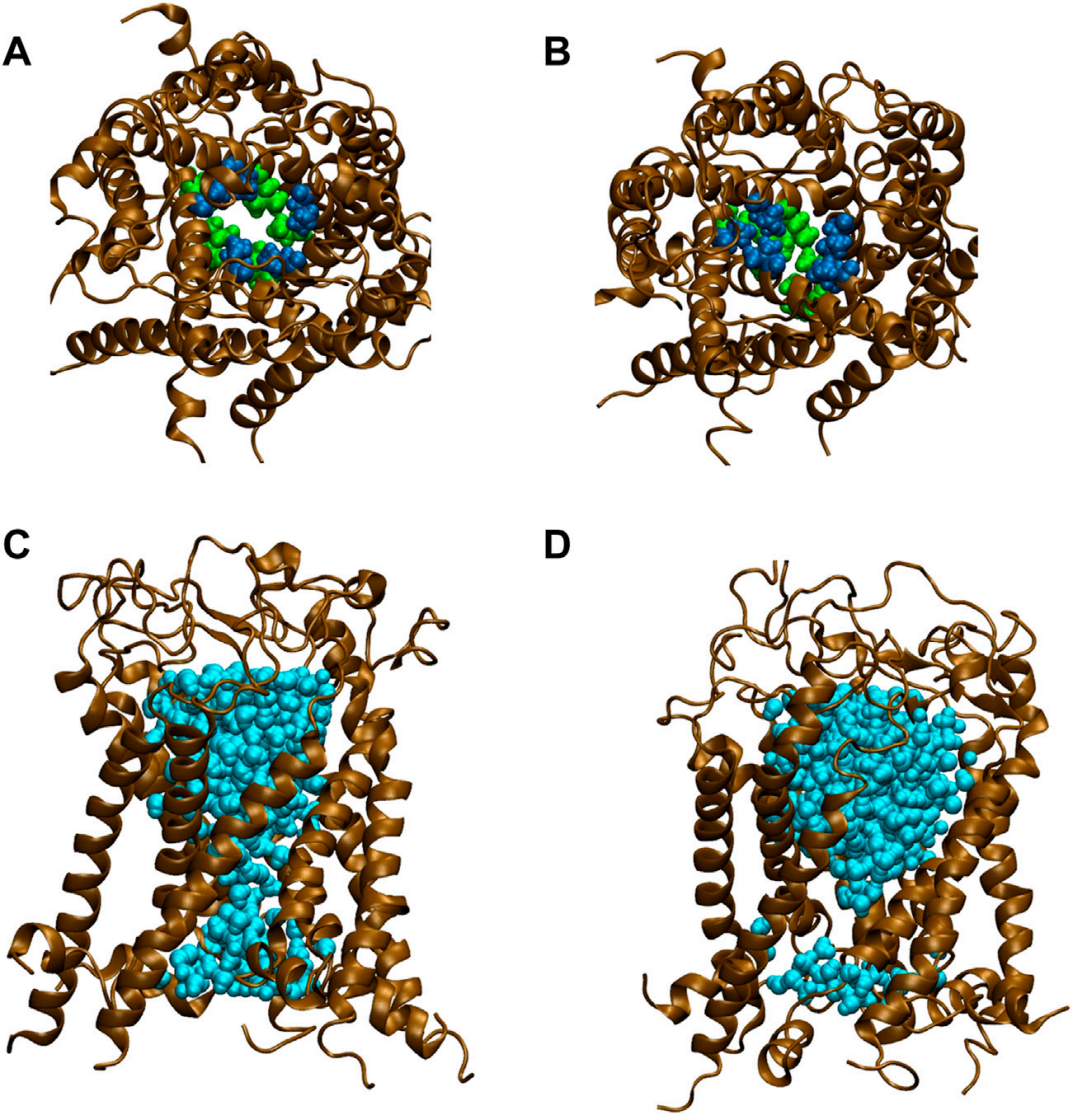
Snapshots of top views and side views focusing on water penetration into the gate region. **(A,C)** top and side views of G22N MscL and **(B,D)** top and side views of G22N/F78N MscL during equilibrium simulations. In the side views, only one TM1 helix located at the front-side is not shown and two amino acid residues of Leu19 (green) and Val23 (blue) are depicted. Lipids and ions are not shown here. Water molecules in the pore were depicted in sky blue colored VDW representation.

All types of MscLs except G22N MscL remained closed during the entire simulation time. The close hydrophobic packing between Gly22 and Ala20 in the neighboring subunit is formed with hydrophobic interaction based on the knob-into-hole association, and Gly22, and Gly26 fit into a pocket formed by Val16, Leu19, and Ala20 in the neighboring subunit (Sawada et al., 2012). In G22N MscL the gate was slightly expanded while spontaneous water permeation occurred (Figure 4). As an index representing the degree of channel opening, we employed the number of water molecules in the gate region, because definition and estimation of the pore radius is practically very complicated and difficult due to very dynamic and complicated 3D geometry of the gate region of MscL. By contrast, evaluation of water accessibility to the gate region is much easier and reflects directly the permeability of MscL, which can quantitatively be estimated by counting the number of water molecules penetrating into the gate region. Thus, we calculated the number of water molecules penetrating into the gate region as a function of simulation time and the result is shown in Figure 4. In WT and F78N MscLs few water molecules were in the gate region, whereas in G22N MscL, 5–10 water molecules and in G22N/F78N MscL channels, 1–5 water molecules were detected during the equilibrium simulations. The corresponding averaged size of the pore radius around Leu19 is 0.87 Å in WT, 0.68 Å in F78N, 3.57 Å in G22N and 0.96 Å in G22N/F78N, respectively. This is consistent with the results shown in Figures 5A, B, in which the gate of G22N MscL opens to some degree, whereas G22N/F78N does not show any indication of opening during the entire equilibration process. Figures 5C, D show the side views of G22N and G22N/F78N MscL channels, where some water molecules have penetrated into the gate region from the periplasmic side both in G22N and G22N/F78N mutants. However, the behavior of water molecules around the gate is quite different between the G22N and G22N/F78N MscL channels: the gate of G22N MscL is fully hydrated, while one short water string exists only in the periplasmic side of the gate of G22N/F78N mutant during the 50 ns of equilibrium simulation.

### 3.4 Simulations of the MscL channel opening process upon membrane stretch

In order to simulate an opening of the MscL channel by increasing tension in the membrane, we applied a force to generate constant membrane tension (150 dyn/cm = 150 mN/m) in the lipid bilayer after the 50 ns of equilibrium simulations. The 50 ns of simulation time is indeed shorter than the actual time length of MscL opening. Therefore, we have extended the MscL opening simulations by applying higher or lower membrane tension than 150 dyn/cm. Results indicate that structural changes of MscL under these conditions are essentially the same (not shown) as the results obtained during 150 dyn/cm simulation including the pore hydration. Proteins such as MscL can be regarded as viscoelastic materials and their mechanical behaviors can be analyzed by using Voigt model. Structure changes of MscL upon membrane stretch correspond to creeping, where in the initial phase of mechanical response, most of applied force is balanced by friction of dashpot, resulting in a very small creeping. MD simulations of several ns reflect only very initial transient creeping towards a final stage. The effect of the amplitude of applied force on MscL creeping during such a short period is mainly reflected in the time course of creeping.

**FIGURE 6:**
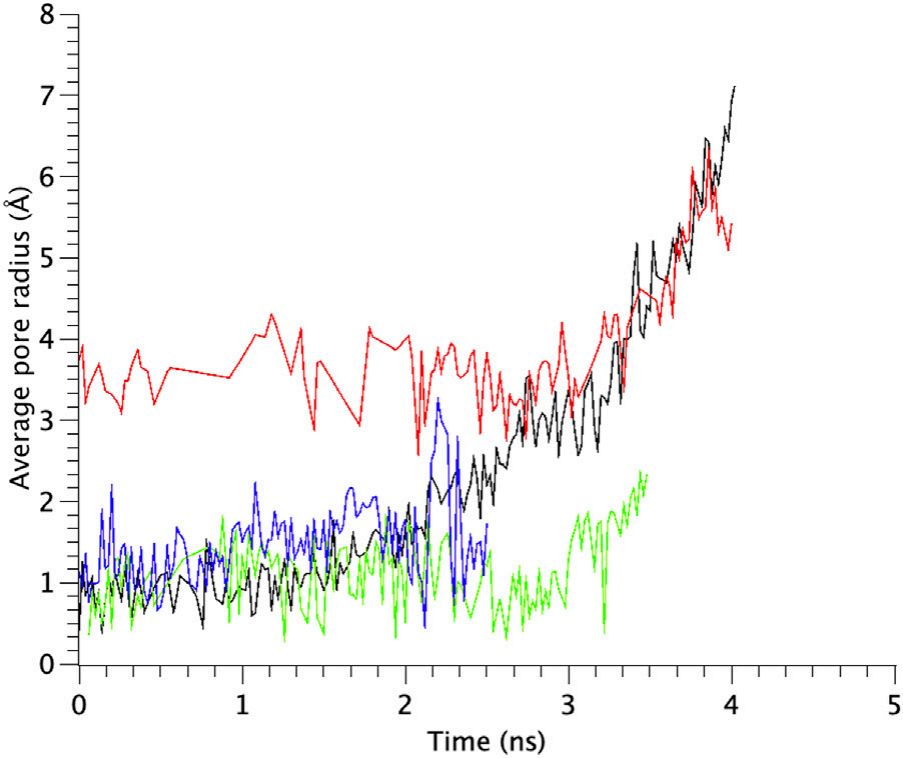
Time courses of change in the average pore radius at the gate region of WT, G22N, F78N and G22N/F78N MscLs in response to tension increase, shown as black-, red-, green- and blue-colored lines, respectively. The average pore size is identified as that around the most constricted part of Leu19.

TM1 and TM2 helices of the WT MscL were gradually tilted and pulled in radial direction, leading to an expansion of the gate. Upon membrane stretch, the thickness of the membrane is gradually decreased. During the decrease of the membrane thickness, both TM1 and TM2 helices tilt accordingly to adjust their length to the hydrophobic mismatch. Consequently, the relative position of Phe78 to the membrane is not changed. This opening behavior is consistent with the previous MD simulations (Sawada et al., 2012) and experimental results (Perozo et al., 2002; Wang Y et al., 2014). In addition to WT MscL simulation, we performed opening simulations with the G22N, F78N and G22N/F78N MscL channels under the same condition. In order to examine the difference in the opening behavior, we used HOLE program and calculated an average pore radius around Leu19 in the gate region of the WT and the mutants of MscL (Figure 6). The size of the narrowest pore constriction at Leu19 in G22N had a much larger initial value, and increased with time as in WT, whereas the pore in F78N and G22N/F78N remained much smaller throughout the simulations. These results are consistent with not only experimental results in this study but also with the previous patch clamp and simulation studies (Yoshimura et al., 1999; Yoshimura et al., 2004; Sawada et al., 2012).

**FIGURE 7:**
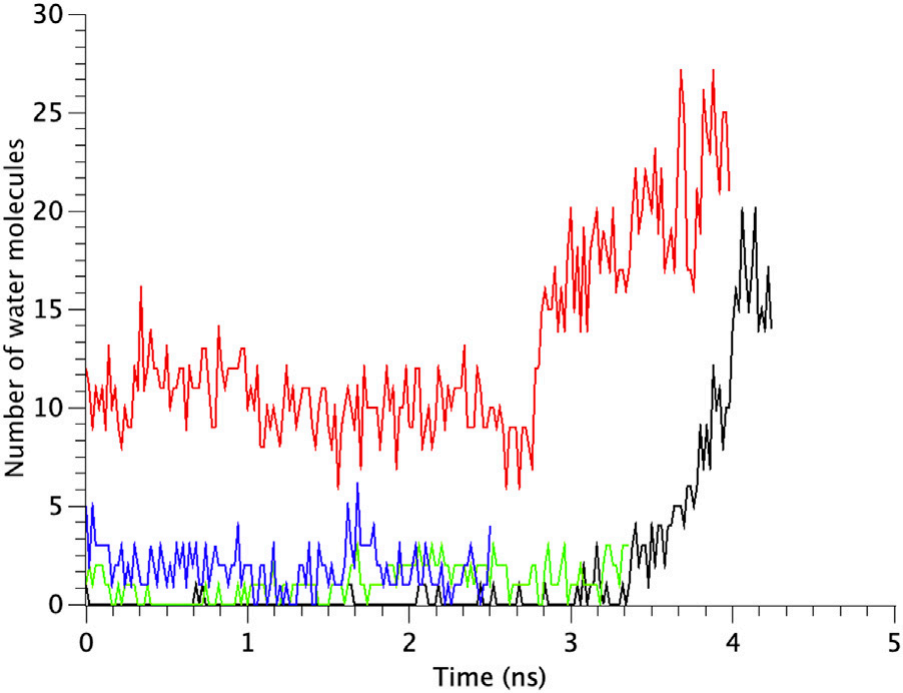
Time courses of the change in the number of water molecules at the gate region of WT, G22N, F78N and G22N/F78N MscLs in response to tension increase, shown as black-, red-, green- and blue-colored lines, respectively. The water molecules are identified as those in the most constricted part of the pore formed by amino acids Leu19 to Val23.

The number of water molecules in the constricted gate region were counted in each type of the MscL channel investigated in our study. Figure 7 shows the time course of changes in the number of water molecules in the gate region of MscL during opening process. The number of water molecules in the gate region increases gradually after 3.0 ns in WT, while that in F78N MscL did not increase and at most a few water molecules are present during 5 ns simulations. In G22N, the gate region is already occupied by ca.10 water molecules in the first 2.6 ns and an increase in the number of water molecules can be observed in the following 1.4 ns. In G22N/F78N MscL, a few water molecules are present within the gate region, but the number of water molecules did not increase during membrane stretch.

## 4 Discussion

One of the core biophysical questions concerning mechanosensitive channels is to understand the mechanism how force-from-lipids (membrane tension) leads to the gate opening, that is, to elucidate biophysical mechanisms how the received force at the tension sensor is transduced to the gate and leads to the channel opening (Teng et al., 2015; Martinac and Kung, 2022). In this study, we approached this question by focusing on the mechano-gating in the bacterial MS channel MscL, because this is one of the best studied MS channels in terms of the structure-function relationship in mechano-gating. It has been suggested that one of the major MscL tension sensors is the Phe78 residue on TM2 helix facing the lipid bilayer (Sawada et al., 2012), and that Gly22 in TM1 lining the channel pore is one of the key components of the gate (Yoshimura et al., 2004). However, the force transmission pathway from Phe78 in TM2 to the gate region in TM1 remained obscure. In order to identify this pathway underlying the opening process we performed patch clamp and MD analyses of the mechano-gating process in the WT and three types of MscL mutants at the sensor (F78N), the gate (G22N), and their combination (G22N/F78N), respectively. Based on the analysis of the difference in the spontaneous channel openings between G22N and G22N/F78N mutants, we found a novel force transmission pathway from the tension sensor Phe78 to the gate corresponding to hydrophobic interaction between Phe78 of the TM2 helix and Ile32-Leu36-Ile40 residues of the TM1 helix of a neighboring subunit.

### 4.1 Major tension sensors in MscL channel

MscL is gated exclusively by tension in the membrane lipid bilayer. Theoretically all the amino acid residues that can interact with membrane lipids could be potential tension sensors. To identify major tension sensor(s), almost all amino acid residues that can face lipids in the periplasmic side of TM1 and TM2 were substituted with Asn and subjected to *in vitro* (patch clamp) and *in vivo* (hypoosmotic-shock) assay and concluded that Leu36, Ile40, and Ile41 (TM1) and Phe78, Ile79, Phe83, and Ile87 (TM2) were the “high impact residues” in terms of tension sensing (Yoshimura et al., 2004). Among them F78N exhibited the severest LOF phenotype. Furthermore, in our MD simulation study (Sawada et al., 2012), we calculated the interaction energy between each amino acid residue in the periplasmic side of TM2 and membrane lipids and found that only Phe78 showed specifically stable interaction with lipids both in the absence and presence of membrane stretch. Thus we conclude that F78 is the major tension sensor in the MscL gating process triggered by membrane stretch. This conclusion seems reasonable considering the specific position of the residue at the rim of the funnel like structure of MscL where the maximal tension is generated in the membrane.

More recently, it has been reported that the amphipathic N-terminal region (S1 domain) tightly interacts with lipids at the cytoplasmic side of the membrane and plays a crucial role in MscL opening (Iscla et al., 2008; Iscla and Blount, 2012; Bavi et al., 2016). Consequently, MscL senses membrane tension both at the cytoplasmic and periplasmic sides of the membrane. The tension-dependent force-from-lipids (Teng et al., 2015; Martinac and Kung, 2022) may act on both Phe78 and the N-terminal domain in opposite directions (Figure 1D), contributing to the opening of the channel gate in a cooperative manner, thus leading to tilting of transmembrane helices and MscL opening. The N-terminal domain is linked to the gate region within TM1 via Gly14, enabling the force to act on the gate directly. Importantly, two phenylalanine residues (Phe7 and Phe10) are conserved in S1 domain. As hydrophobic residues, they have high affinity to membrane lipids and thus support anchoring of the amphipathic N-terminal domain in the inner leaflet of the membrane bilayer, which is essential for pulling the N-terminal helix by membrane tension (Iscla et al., 2008; Iscla and Blount, 2012; Bavi et al., 2016).

### 4.2 Unexpected results from electrophysiological experiment on the double mutant G22N/F78N

Concerning the channel opening of MscL, it has been generally accepted that the MscL pore expands through tilting of inner (TM1) and outer (TM2) helices while TM1 helices are sliding against each other, in an iris like movement leading to the channel opening upon membrane stretch. Earlier studies of the Asn mutation at the gate (G22N) showed that this MscL mutation underwent spontaneous channel openings in the absence of membrane stretch (Yoshimura et al., 1999; Yoshimura et al., 2008) (Figure 2A). In agreement with this finding, our previous simulation study demonstrated that the gate region in G22N MscL contains water molecules even in the absence of membrane stretch, leading to spontaneous ion and water permeation (Sawada et al., 2012) (Figure 5C). This is because hydrophilic side chain of the substituted asparagine (Asn) in G22N MscL attracts water molecules to the gate region, leading to a formation of a chain of water molecules, which contribute to spontaneous ion and water permeation by breaking the vapor lock (dewetted to wetted transition).

By contrast, MscL with a point mutation at the proposed tension sensor (F78N) did not open even at a large negative pressure applied to the pipette (Figure 3B). In order to check that the apparent total loss of function of F78N is not due to abnormal membrane trafficking, we measured protein expression levels of F78N on the plasma membrane by Western blot analysis and confirmed that the presence of F78N in the membrane (Yoshimura et al., 2004). In F78N mutant channel, the interaction between Asn78 and membrane lipids is not dramatically different from that between Phe78 and lipids in WT channel. However, in the F78N, Asn78-water interaction is evidently stronger than Asn78-lipid interaction, and when the membrane is stretched, there is high probability for water penetration into the gap between Asn78 and lipids. Actually our previous simulation study (Sawada et al., 2012) showed that Asn was completely decoupled lipids by penetrating water molecules under membrane stretch. We believe that this is the reason why F78N shows such a severe LOF phenotype.

We initially speculated that the double mutation (G22N/F78N) would not affect the spontaneous channel opening without membrane stretch, because it might occur independently of the tension sensor, we thought. However, results showed that not only stretch dependent activation (Figure 3Ci) but also spontaneous channel openings (Figure 3Cii) were absent in the double mutant MscL.

This result indicates that spontaneous channel opening is not independent of physicochemical property of the tension sensing site Phe78. To elucidate how and to what extent Phe78 contributes to the mechanisms of the force transduction from this site in TM2 to the gate region lined by TM1, we examined a possible mechanism as discussed in the following sections.

### 4.3 Different behaviors between G22N and G22N/F78N in terms of spontaneous channel opening

TM1 helix interacts with the neighboring TM1 helices *via* hydrophobic amino acids including Leu19, Gly22 and Val23, which form the gate at the cytoplasmic side of the bilayer. Closed state of the gate is stabilized by this hydrophobic interaction (hydrophobic lock) to prevent water/ion permeation, which is blocked by the hydrophobic nature of the gate constituting amino acids, including Gly22 and the surrounding amino acid residues Leu19 and Val23 (vapor lock by dewetting) (Sawada and Sokabe, 2015). Spontaneous channel openings observed in G22N is induced through the instability of the hydrophobic/vapor lock by the replacement of hydrophobic Gly22 with hydrophilic Asn22. Since Asn22 has a larger side chain than Gly22, it is conceivable that Asn22 experiences steric hindrance due to its proximity to the neighboring Val23. Thus, the size of the narrowest part of the pore at Leu19 in G22N MscL increases after 50 ns equilibration as shown in Table 1. By contrast WT as well as F78N with Gly22 showed no such expansion at Leu19 (Table 1). Likely, the physicochemical properties of Asn22 destabilize the hydrophobic/vapor lock at the gate to induce spontaneous gate opening and water permeation.

**TABLE 1:** Summary of the interaction energy between the amino acid residue Phe78 and lipids and between the amino acid residue Gly22 and water, changes in the size of the pore at Leu19 (∆rLeu19) and Ile40 (∆rIle40) and the tilt angle change of TM1 helix in each type (WT, G22N, F78N and G22N/F78N) of MscLs during equilibration. The change in both the size of the pore and in the tilt angle (mean ± standard error) are calculated based on the difference in the coordinates between at the initial and at 50 ns of equilibrium simulation.

Type of MscL	Interaction energy between the amino acid residue 78 and lipids (kcal/mol)	Interaction energy between the amino acid residue 22 and water (kcal/mol)	∆rLeu19 (Å)	∆rIle40 (Å)	Tilt angle change (degree) (n = 25)
WT	−43.11	−9.75	−0.1	0	−0.7 ± 0.7
G22N	−40.29	−53.95	1	1.5	−0.4 ± 0.7
F78N	−39.75	−7.18	−0.3	0.5	2.3 ± 0.5
G22N/F78N	−41.36	−48.58	−0.3	1.3	5.1 ± 0.9

The double mutant (G22N/F78N) showed neither gate expansion nor spontaneous water permeation as shown in Figure 5. In order to understand why G22N and G22N/F78N behave differently during equilibrating process, we analyzed tilting motion of TM1 helices, because helix tilting is one of the indices for the initiation of gate opening (Sawada et al., 2012). The right-most column in Table 1 shows changes in the tilt angles of TM1 helices for all types after 50 ns of the equilibration process. Results indicate that tilt angles of the TM1 helix in G22N and WT MscL show no significant difference, with the TM1 helices tilting towards the membrane plane by 2.3 (F78N) and 5.1 (G22N/F78N) degrees, respectively. Substitution of Phe78 with Asn78 seems to cause a conformational change around this position, leading to specific tilting motion, which inhibits the spontaneous gate opening. Actually, the tilting decreases the size of the gate as described below.

### 4.4 Transmission of the force sensed by Phe78 to the MscL gate

The gate region of MscL is constituted of hydrophobic amino acid residues. Therefore, penetration of water in the hydrophobic gate region is energetically unfavorable during the closed state by the mechanism called hydrophobic “vapor lock” (Anishkin and Sukharev, 2004; Sawada and Sokabe, 2015). However, G22N MscL allows spontaneous water permeation through the gate even during the equilibration process, whereas G22N/F78N MscL exhibited no permeation of water except for the water penetration into the upper portion of the gate, as shown in Figures 5C, D. Apparently, replacement of Phe78 with Asn78 causes this difference most likely due to the hydrophilicity of the Asn residue, which impairs the hydrophobic interaction between the lipid bilayer and the residue at the 78 site of the TM2 helix, as mentioned above. However, given that the interaction of Asn78 with lipids is approximately as strong as that of Phe78 in the G22N mutant under no external membrane stretch (Sawada et al., 2012; Table 1 in this study), it was prudent to explore why spontaneous channel openings observed in G22N (Figure 2A) were inhibited in G22N/F78N mutant (Figure 3Cii).

The first possible mechanism to consider could involve the periplasmic loop that links the TM1 and TM2 helices. This is a straightforward mechanism, because the force sensed at Phe78 in TM2 may be directly conveyed to TM1 helix in the same subunit. However, considering the very flexible nature of the loop, this is not energetically efficient way to transmit the force from the Phe78 tension sensor in TM2 to the gate in TM1.

The second possible mechanism for the force transmission could be *via* helix-helix interaction between two neighboring MscL subunits. As indicated previously, Lys31 in one subunit forms a salt bridge with Asp84 in the neighboring subunit (Gullingsrud and Shulten, 2003). Therefore, we calculated the interaction energy between these two amino acid residues in all types of the MscL channels studied here and found that no obvious differences were observed between WT and mutant MscLs. This result clearly indicates that any mutations employed in this study do not affect the strength of the salt bridge between the neighboring subunits in the three MscL channels. The salt bridge seems to be critical for forming stable close packing between the TM1 and the neighboring TM2 helices and therefore it may be a candidate for the force transmission pathway. However, since it is not affected by the mutations, the salt bridge cannot be the origin of the drastic change in the gating behavior of GOF mutant of MscL (G22N). From the structural point of view, another helix-helix interaction between Val33-Leu36 in TM1 and Ile87 in TM2 within the same subunit could be a mechanism for the force transmission because these three amino acid residues are close to each other. However, the calculated interaction energy between Val33-Leu36 and Ile87 is not affected by the substitution of Phe78 for Asn22 (data not shown).

**FIGURE 8:**
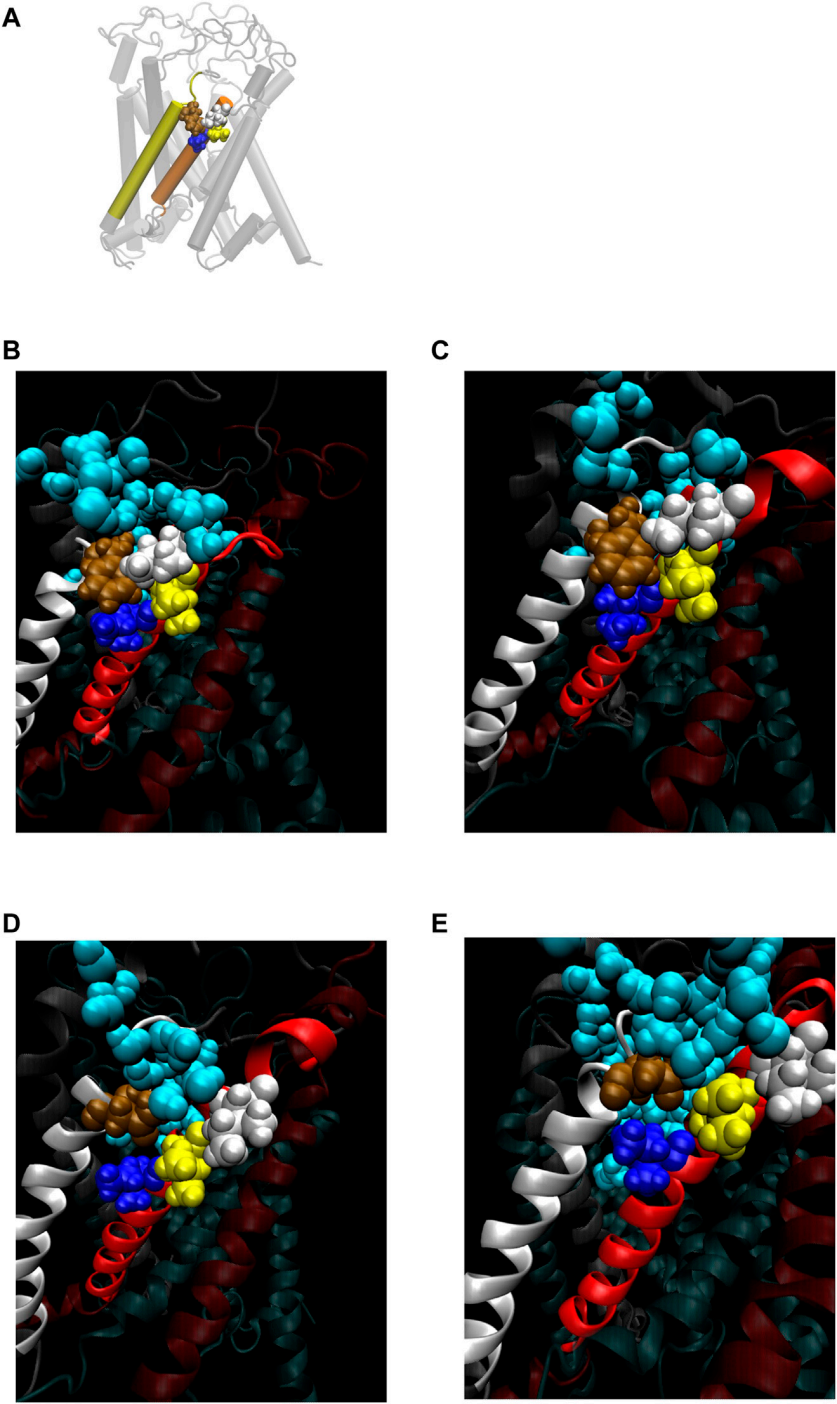
**(A)** Cartoon representation of MscL with one pair of Ile32-Leu36-Ile40 and Phe78 highlighted in blue, yellow, white and brown colored VDW representation, respectively. **(B–E)** Snapshots of the configuration focusing on water penetration around the amino acid residue 78 in WT **(B)**, G22N **(C)**, F78N **(D)** and G22N/F78N **(E)** MscL during equilibrium simulation. In all snapshots, one TM1 helix and the neighboring TM2 helix are shown in a ribbon representation with different colors (TM1: red, TM2: white), where Ile32, Leu36, Ile40, the amino acid residue 78 (Phe78 in WT and G22N, Asn78 in F78N and G22N/F78N) and water molecules around the TM1-TM2 contact are depicted in blue, yellow, white, brown and sky blue colored VDW representations, respectively.

**FIGURE 9:**
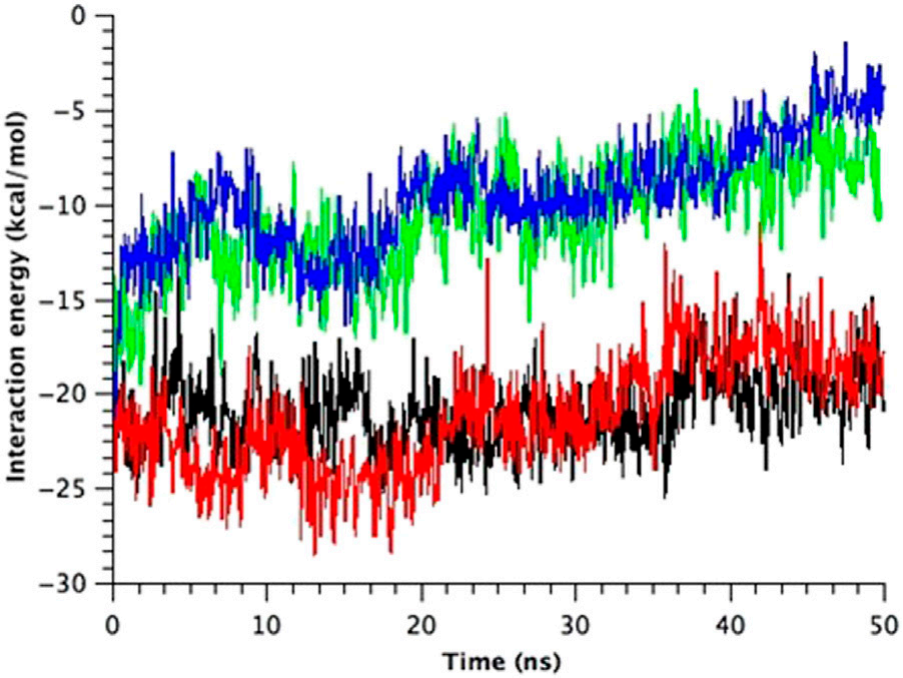
Time course change in the total interaction energy summed up from five interactions between Ile32-Leu36-Ile40 in one TM1 helix and Phe78 in the neighboring TM2 helix in the equilibrium simulations. The interaction energy for each model of MscL is depicted in black (WT), red (G22N), green (F78N) and blue (G22N/F78N) color, respectively.

Finally, there is a possibility of the third force transmission mechanism *via* the interaction between Ile32-Leu36-Ile40 in TM1 and Phe78 in TM2 in the neighboring subunit. The interaction between Phe78 and Leu36 and Ile40 in the neighboring subunit has been reported in the previous work (Maurer and Fougherty, 2003). As shown in Figure 8, WT and G22N MscLs demonstrate that Phe78 in TM2 of the a subunit interacts with Ile32, Leu36 and Ile40 in TM1 of the neighboring subunit, whereas F78N and G22N/F78N MscLs show water molecules penetrating into the gap between Asn78 and neighboring the TM1 residues. In addition, these three amino acid residues in TM1 helix do not interact with Asn78 efficiently (Figure 8). Figure 9 shows the interaction energy profiles between Ile32-Leu36-Ile40 in one subunit and the neighboring Phe78 or Asn78 in the closed state. A striking feature is that the interaction energy in WT and G22N MscLs has similar values (−23 kcal/mol) and the interaction was maintained during the closed state, whereas in F78N and G22N/F78N MscLs, the interaction energies show similar values like in WT (or G22N), (i.e., −13 kcal/mol) during the first 5 ns, and then gradually decreased to finally reach −7kcal/mol after 40 ns of the equilibration process. Thus, we conclude that mechanical decoupling in the force transmission pathway occurs in F78N and G22N/F78N mutants, whose influence on the MscL gating is considered in detail in the following sections.

### 4.5 Tight interaction between TM1 and TM2 around Phe78 is important for the channel opening

**FIGURE 10:**
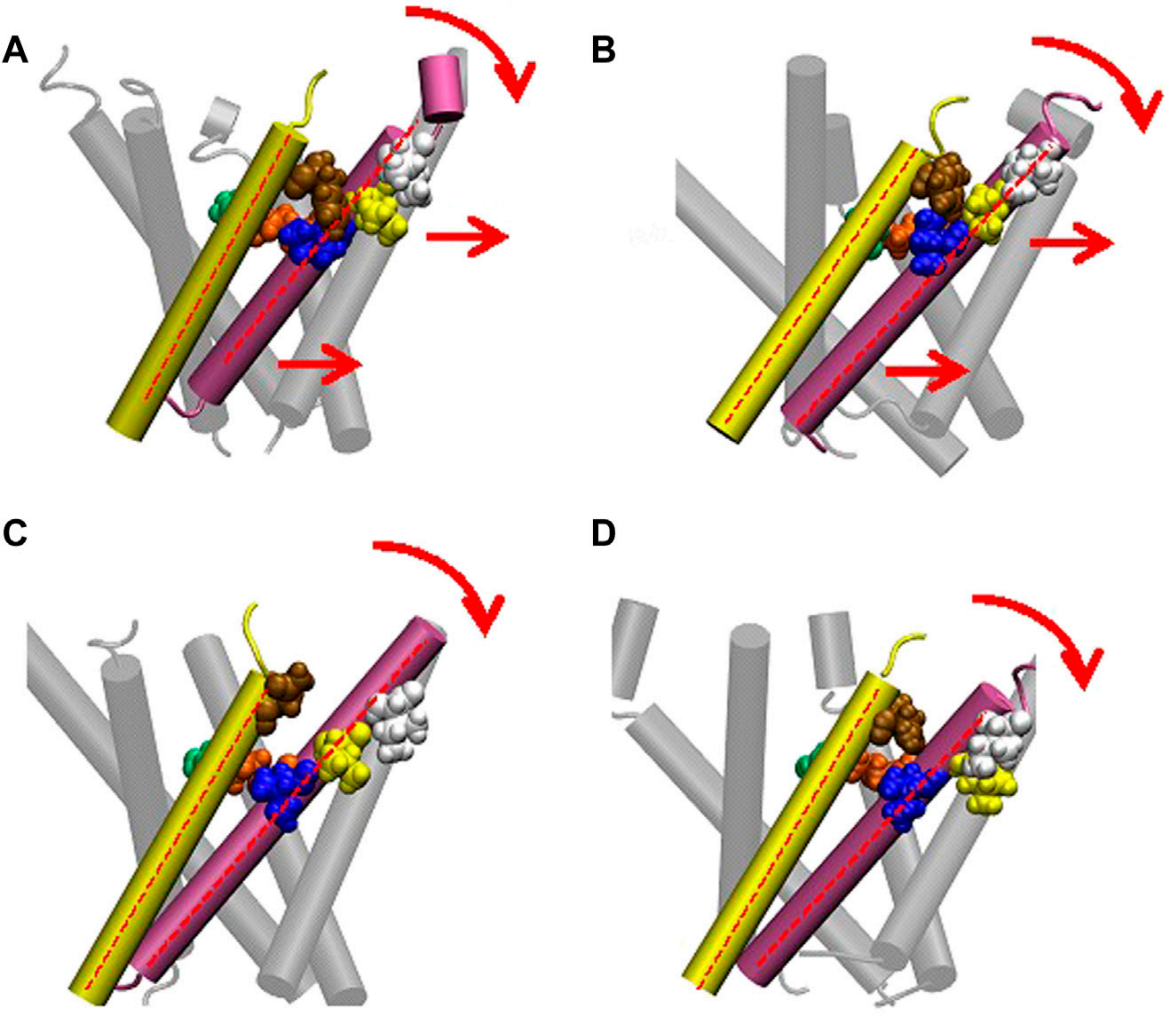
Cartoon representation of MscL and one pair of Ile32-Leu36-Ile40 and Phe78 is highlighted in blue, yellow, white and brown colored VDW representation in WT **(A)**, G22N **(B)**, F78N **(C)** and G22N/F78N **(D)**. Red colored dotted lines indicate the axis of transmembrane helices.

We made a detailed analysis of the tilting motion of the TM1 and TM2 helices observed in G22N/F78N MscL. It has been reported that Lys31 in one TM1 and Asp84 in the neighboring TM2 helix form strong electrostatic interaction (Gullingsrud and Shulten, 2003). We found that during TM1 tilting in G22N/F78N MscLs Lys31 works as a pivot point. Because of the hydrophilic nature of Asn78, this residue can interact not only with lipids but also with water molecules. Consequently, when membrane is stretched, water molecules penetrate the gap between Asn78 and Ile32-Leu36-Ile40 and decouple the original interaction between Phe78 and Ile32-Leu36-Ile40**.** Due to the penetration of water into the gap between Asn78 and Ile32-Leu36-Ile40 observed both in F78N and G22N/F78N, the periplasmic side of the TM1 helix weakens the interaction between TM1 and TM2, leading to instability of the TM1 original position in the closed state (Figure 10). Thus, as shown in Figures 10C, D, TM1 of F78N and G22N/F78N MscLs tilt spontaneously pivoting at Lys31. When TM1s tilt, they are in a close apposition to each other at Leu19, leading to narrowing of the pore size at the gate. By contrast, in WT and G22N the contact between Phe78 and Ile32-Leu36-Ile40 of the neighboring TM1 helices is stable, and TM1 helix, like a rigid rod supported by two fixed points, does not undergo spontaneous tilting (Figures 10A, B). As shown in Table 1, the size of the cytoplasmic side of the pore at Leu19 becomes slightly smaller at 50 ns after the onset of equilibration in both F78N and G22N/F78N MscLs. It is suggested that both F78N and G22N/F78N MscLs cannot maintain the close contact between Asn78 and Ile32-Leu36-Ile40 due to penetration of water molecules into the gap between Asn78 and Ile32-Leu36-Ile40. This consequently leads to tilting of the TM1 helix toward the membrane plane by pivoting around Lys31 driven by the resting tension in the membrane bilayer. The gate region eventually becomes narrower, which prevents spontaneous water permeation across the gate. The size of the periplasmic side of the pore at Ile40 of the G22N/F78N mutant becomes a bit larger compared to that of F78N, probably because of the relatively easier movements of Asn22 due to its unstable positioning surrounded by hydrophobic amino acids at the gate.

### 4.6 Behavior of WT and mutant MscLs in response to membrane stretch

In this study, we found a novel link for transduction pathway from the sensing the mechanical force at Phe78 into the opening of the MscL channel. The link is provided by the stable hydrophobic interaction between Phe78 in the TM2 helix and Ile32-Leu36-Ile40 in the TM1 helix of the neighboring subunit of the channel. When Phe78 was substituted with Asn as in the F78N and G22N/F78N MscL mutant channels, the substituted amino acid Asn78 could not maintain stable interactions with Ile32-Leu36-Ile40 amino acid residues, while the strong electrostatic interaction between Lys31 and Asp84 was maintained as in the WT and G22N channels. However, only with this single linkage between TM2 and neighboring TM1, the TM1 helices of the F78N and G22N/F78N mutant channels were not efficiently pulled on by the interacting TM2 helices. More importantly, Asn78 was not pulled on sufficiently by the facing lipids either. Because of the hydrophilic nature of Asn78, it can interact not only with lipids but also with water molecules, and therefore, when membrane is stretched, water molecules penetrate the gap between Asn78 and Ile32-Leu36-Ile40, which cause decoupling of the original interaction between Phe78 and Ile32-Leu36-Ile40. As a result, both F78N and G22N/F78N MscL mutants behaved like loss-of-function (LOF) mutants as we consistently observed both in experimental and MD simulation studies in this report.

In a separate study, the N-terminal amphipathic helix of MscL was found to act as a crucial structural element during the channel gating induced by membrane tension by coupling the channel to the lipid bilayer (Iscla et al., 2008; Iscla and Blount, 2012; Bavi et al., 2016). To consolidate the finding about Phe78 mechanosensor in this study with the reported role of the N-terminal domain in MscL gating, we propose that both the Phe78 residue and the N-terminal helix act as mechanosensors complementing each other’s role in the MscL opening by membrane tension. During the opening process following the tilting of both TM1 and TM2 helices, membrane tension sensed at Phe78 at the periplasmic side of TM2 pulls on TM1 *via* the Ile32-Leu36-Ile40 interaction with Phe78 in the direction of the force acting on the TM2 helix at the periplasmic side of the channel while tension acting on the N-terminal region at the cytoplasmic side pulls on the TM1 helix in the opposite direction (Figures 1D, Figure 10). If our hypothesis postulating the hydrophobic interactions between Phe78 and Ile32-Leu36-Ile40 is essential for proper tilting of TM1 helix due to the force pulling on TM2 is correct, it is not surprising that interaction of the three hydrophobic amino acid residues with a hydrophilic amino acid residue like Asn would result in channels behaving like LOF mutants. Significantly, we have already reported that I32N, L36N and I40N MscL mutant channels are all LOF mutants, the reason for which remained unclear at the time of publication of these results (Yoshimura et al., 2004). Consequently, any one of the four amino acid residues, i.e., Ile32, Leu36, Ile40, and Phe78, is likely to be essential for coupling the force transmission from TM2 to TM1 and together with the force acting on the N-terminal helix contributing to mechano-gating in the WT MscL channel.

## 4 Conclusion

This study helped us to elucidate the essential role of Phe78 in the MscL gating by the force-from-lipids. The most important finding here is the identification of a novel force transduction pathway from the tension sensing at Phe78 within the TM2 to the channel opening through the interaction with Ile32-Leu36-Ile40 in the neighboring TM1 helix. In addition, a stable salt bridge between Lys31 in TM1 and Asp84 in the neighboring TM2 was found to contribute to stabilizing the closed structure of MscL, working as a pivot and a force transmission point from TM2 to the neighboring TM1 helix. Thus, MscL has two important force transmission points, Lys31 = Asp84 and Phe78 = Ile32-Leu36-Ile40, from the outer TM2 helix to inner TM1 helix, and Gly14 coupling the force acting on the N-terminal helix directly to the gate. These force transmission points synchronize the movement of both helices during the MscL channel opening by membrane tension.

**FIGURE 11:**
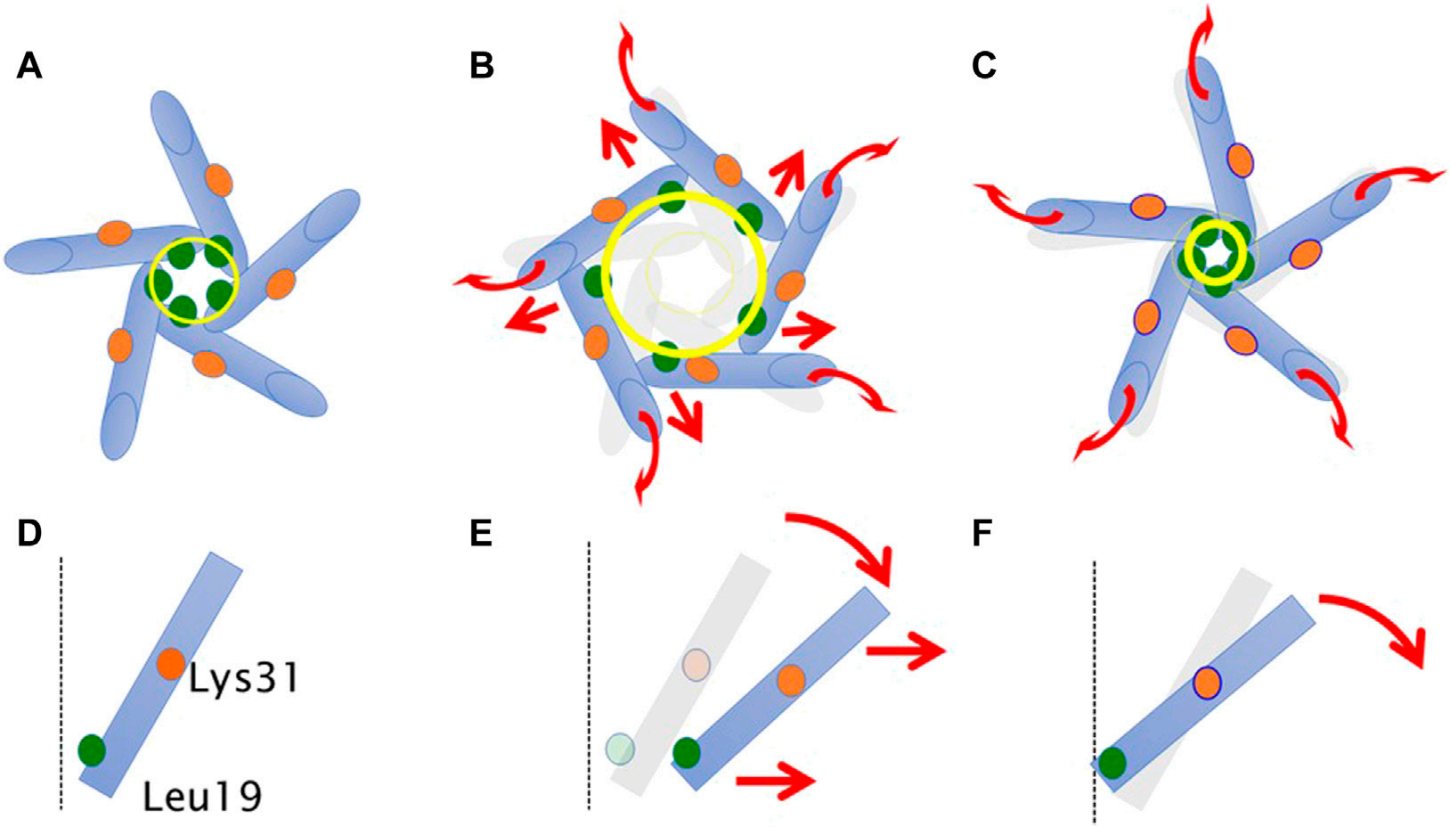
Schematic representation showing a motion of transmembrane helices of MscL during equilibration. Shown are top **(A–C)** and side (a single TM1 helix is depicted) **(D–F)** views of the TM1 α-helix (*cylinder*) in WT **(A,D)**, G22N **(B,E)**, F78N and G22N/F78N **(C,F)**. Leu19 is located at the cytoplasmic half of TM1 (*green colored circle*). Lys31 shown as orange colored circle is located at the middle of TM1. The size of the gate region is depicted as yellow colored circle (solid line: after equilibration, transparent line: at the beginning). Gray colored representation of transmembrane helices represents its original position at the beginning of equilibration. Dotted black colored vertical line represents the channel axis along *z*-axis. Red arrows represent directions of motion of TM1 helices during equilibration. This schematic representation is quantitatively consistent with the results from MD calculations, and the shift and the tilting motions are described based on the measurements of the change in the coordinates.

Based on the above findings, we summarize the gating behavior of the WT and each MscL mutant investigated here under no membrane stretch. WT MscL senses membrane tension at the amino acid residues facing to the membrane lipids in TM2 and the Phe78 residue mainly acts as a critical tension sensor, but the WT MscL remains in the closed state (Figures 11A, D). The gain of function (GOF) mutant G22N MscL causes a slight expansion of the gate (Figures 11B, E) and wetting of the gate, which results in spontaneous channel openings. Further introduction of F78N mutation in the G22N mutant channel decouples the Phe78 = Ile32-Leu36-Ile40 interaction, which together with the membrane tension pulling on the N-terminal helix leads to tilting of the TM1 helix around a single fixed point of MscL. (Lys31-Asp84) (Figure 11F). This motion results in a decrease of the size of the channel pore at the gate in the resting state (Table 1; Figure 11C) and loss of spontaneous channel openings.

In conclusion, WT MscL opening is regulated by a complementary action of the Phe78 residue and the N-terminal amphipathic helix. Phe78 acts as a tension sensor and a force transmitting residue from the TM2 to the TM1 helix in the neighboring subunit through the Phe78 = Ile32-Leu36-Ile40 interaction, which is coordinated with membrane tension pulling on the N-terminal helix and causing the TM1 helix to tilt. These two mechanosensing elements within the MscL channel structure are thus pulled by the force-from-lipids in opposite directions, which results in the tilt of all helices towards the membrane plane and enabling the iris-like opening of the MscL channel.

## Data Availability

The raw data supporting the conclusion of this article will be made available by the authors, without undue reservation.
